# Reduction of Kinematic Short Baseline Multipath Effects Based on Multipath Hemispherical Map

**DOI:** 10.3390/s16101677

**Published:** 2016-10-12

**Authors:** Miaomiao Cai, Wen Chen, Danan Dong, Le Song, Minghua Wang, Zhiren Wang, Feng Zhou, Zhengqi Zheng, Chao Yu

**Affiliations:** 1 Engineering Center of SHMEC for Space Information and GNSS, East China Normal University, Shanghai 200241, China; mmcai@outlook.com (M.C.); dndong@cs.ecnu.edu.cn (D.D.); songlele1989@163.com (L.S.); wangwangzhiren@163.com (Z.W.); zhoufecnu@163.com (F.Z.); zqzheng@ee.ecnu.edu.cn (Z.Z.); cyu@sist.ecnu.edu.cn (C.Y.); 2 Shanghai Key Laboratory of Multidimensional Information Processing, East China Normal University, Shanghai 200241, China; 3 College of Surveying and Geo-Informatics, Tongji University, Shanghai 200092, China; wmhua@126.com

**Keywords:** GPS, multipath hemispherical map, multipath mitigation, kinematic shipborne environment

## Abstract

Multipath hemispherical map (MHM) is a kind of multipath mitigation approach that takes advantage of the spatial repeatability of the multipath effect under an unchanged environment. This approach is not only suitable for static environments, but also for some kinematic platforms, such as a moving ship and airplane, where the dominant multipath effects come from the platform itself and the multipath effects from the surrounding environment are considered minor or negligible. Previous studies have verified the feasibility of the MHM approach in static environments. In this study, we expanded the MHM approach to a kinematic shipborne environment. Both static and kinematic tests were carried out to demonstrate the feasibility of the MHM approach. The results indicate that, after MHM multipath mitigation, the root mean square (RMS) of baseline length deviations are reduced by 10.47% and 10.57%, and the RMS of residual values are reduced by 39.89% and 21.91% for the static and kinematic tests, respectively. Power spectrum analysis has shown that the MHM approach is more effective in mitigating multipath in low-frequency bands; the high-frequency multipath effects still exist, and are indistinguishable from observation noise. Taking the observation noise into account, the residual reductions increase to 41.68% and 24.51% in static and kinematic tests, respectively. To further improve the performance of MHM for kinematic platforms, we also analyzed the influence of spatial coverage and resolution on residual reduction.

## 1. Introduction

The applications of global navigation satellite system (GNSS) technology have expanded rapidly from precise positioning to attitude determination, and from static to kinematic. For attitude determination, the errors from orbit, ionosphere, and troposphere are basically eliminated using a short baseline solution. The multipath effect becomes one of the primary error sources, because it lacks correlation between the ends of the baseline and it is hard to parameterize for estimation.

Scientists put great efforts towards mitigation of the multipath effect from hardware-based and software-based aspects. Among various software-based approaches, one category makes use of time series analysis, such as the weighted adjustment based on observed signal-to-noise ratio (SNR) [1,2,3], and filtering techniques including wavelet analysis [4,5,6,7], adaptive finite-duration impulse response (FIR) filter [8], and the Vondark filter with cross-validation [9]. These approaches are time series-dependent, and are hence more suitable for postprocessing. The second category relates the residual observables (pseudorange, carrier phase, and signal-to-noise ratio) with multipath parameters based on functional models. The parametric estimation approach minimizes the adjustment parameters by utilizing an external atomic clock to connect multiple receivers and forms single-differences for short baselines to estimate the multipath parameters [10]. This approach has the potential to mitigate multipath effect in real-time for moving platforms. However, it requires noisy pseudorange and signal-to-noise ratio observables for joint inversion, and the results rely on the functional model linking the L2 and P2 multipath effect. The third category takes advantage of the intrinsic spatiotemporal repeatability of multipath effect when the environment is unchanged. Sidereal filtering (SF) is based on the repeatability of the multipath effect in the time domain. If the antennas keep static and the environment remains unchanged, the multipath effects of consecutive sidereal days are highly correlated due to the sidereal repeat period of Global Positioning System (GPS) constellation [11,12,13,14,15,16,17]. The SF approach for multipath mitigation has been implemented in precise point positioning [16,18,19], and was applied to natural hazard warning [19,20,21] and structural health monitoring [17,22,23]. However, such a temporal repeatability is no longer viable for a moving platform. The multipath look-up table [24], multipath calibration map [25], empirical site model [26], multipath stacking map [27], and multipath hemispherical map (MHM) [28] are based on the repeatability of the multipath effect in space domain. When the environment is unchanged, the multipath effects are only subjected to the satellite position in the sky, no matter which satellite it is and when the satellite passes the position on the sky. These approaches are certainly suitable for static platforms with unchanged environments for real-time multipath reduction. When the primary multipath effects stem from the platform body itself and the multipath effects from the surrounding environment are minor or negligible, these spatial repeatability-based approaches are also suitable for these moving platforms. For example, the dominant multipath effects of ships and airplanes come from the platform bodies, and the multipath effects from surrounding clouds and ocean waves are considered minor or negligible.

With the increasing applications of kinematic positioning—in particular, the real time kinematic (RTK) technique is utilized widely in navigation and engineering—the multipath effect of a moving platform becomes one of the primary error sources for kinematic positioning. In this paper we investigate the spatial repeatability-based approach for kinematic multipath mitigation, since it is easier to implement in real-time positioning and attitude determination. Cohen et al. [24,29] had demonstrated the feasibility of a spherical harmonic model for multipath reduction for attitude determination of small spacecraft in low earth orbit through a ground-based analog experiment. Although their experiment was enlightening, the spatial resolution of the model was limited to 30° by 30°. In order to reach 1° by 1° resolution, a degree of 360 spherical harmonic model (130321 coefficients) has to be constructed, which is practically difficult to implement in real-time multipath mitigation. We focus on the MHM approach because it can easily reach 1° by 1° spatial resolution and is easier to implement in real-time multipath mitigation. In this paper, we extend the MHM approach to a kinematic shipborne environment. Both static and kinematic shipborne tests were carried out to demonstrate the theoretical feasibility and to further investigate the implementation requirements in real-time and kinematic applications.

## 2. Method

In this section, we first briefly review the principles of MHM, then establish the MHM model for the kinematic shipborne environment and discuss coordinate system transformation.

### 2.1. Theoretical Background of MHM 

For the experiment, we used the Trimble BD982 GNSS receiver (GPS used only), which utilizes the same clock for two antennas. Thus, the single-difference between two antennas is able to simultaneously eliminate the errors of both receiver and satellite clocks. It also eliminates the effects of ionospheric and tropospheric delays due to the short baseline. The single-difference phase observable is expressed as [28]:

(1) $$\Delta \phi^{i}\left(t\right)=\frac{\Delta \rho^{i}\left(t\right)}{\lambda }+\Delta N^{i}+\Delta \phi_{UPD}\left(t\right)+\Delta \phi_{mth}^{i}\left(t\right)+\varepsilon^{i}$$

where the symbol Δ represents the single-difference operator; λ represents the wavelength of the carrier, and here only the L1 observations were used; $\phi^{i}$, $\rho^{i}$, $N^{i}$, $\phi_{mth}^{i}$, and $\varepsilon^{i}$ represent the carrier-phase observable, geometry distance from satellite i to receiver, integer ambiguity, multipath effects, and observation noise, respectively; $\Delta \phi_{UPD}$ represents the differenced uncalibrated phase delay (UPD) between two antennas, which contains the initial fractional phase delay and the propagation delay due to cable and hardware path. Since the UPD is the same for the phase observables from all satellites, the UPD parameter is highly correlated with the ambiguity parameters. The integer part of the UPD parameter is conventionally merged into ambiguity parameters [30], so that $\Delta \phi_{UPD}$ in Equation (1) represents the fractional part of the real UPD.

In our experiment, we used the Kalman filter to process the single-difference observables twice. In the first run, the estimated parameters X contained baseline vectors, float ambiguities, and UPD parameters under the constraint of 0.5 cycle. In the second run, all ambiguities were fixed to integers (all fixed ambiguities had been checked and no abnormal solutions were detected), and the UPD parameter was constrained to its estimated value (obtained from the first run) with the constraint of 0.1 cycle, then the estimated parameters X contained baseline vectors and the tightly constrained UPD parameter. Under such a procedure, the baseline multipath effects basically resided in the residuals. The state transfer equation is expressed as Equation (2) and the observation equation is expressed as Equation (3):

(2) $$X_{k+1}=A_{k}X_{k}+w_{k}$$

(3) $$L_{k}=H_{k}X_{k}+v_{k}$$

where $X_{k}$ represents the estimated parameters at epoch k; A is the state transition matrix that represents the dynamic evolution of the estimated parameters; H is the observation matrix that relates the estimate parameters X to single-difference observable L, and the time-dependent positions of the master antenna in H are calculated using the pseudorange observations of the master antenna; v is the observation noise, which is assumed to be white noise with zero means and covariance matrix $Q_{k}$; w is the state perturbation, which is a Markov stochastic process with covariance matrix $R_{k}$, and $R_{k}=E\left\{w_{k}{w_{k}}^{T}\right\}=r^{2}\Delta t$, r is defined by user. Moreover, w and v are statistically independent.

The state prediction equations of solutions and covariance matrices from epoch $t_{k}$ to $t_{k+1}$ (denoted by k+1|k) and the updated equations are:

(4) $$X_{k+1|k}=A_{k}X_{k}$$

(5) $$C_{k+1|k}=A_{k}C_{k}A_{k}^{T}+R_{k}$$

(6) $$X_{k+1}=X_{k+1|k}+K_{k+1}\left(L_{k+1}-H_{k+1}X_{k+1|k}\right)$$

(7) $$C_{k+1}=C_{k+1|k}-K_{k+1}H_{k+1}C_{k+1|k}$$

where

(8) $$K_{k+1}=C_{k+1|k}H_{k+1}^{T}{\left(Q_{k+1}+H_{k+1}C_{k+1|k}H_{k+1}^{T}\right)}^{-1}$$

is the Kalman gain matrix. The superscript T denotes the transpose of a matrix and C is the parameter covariance matrix.

### 2.2. Implementation of the MHM Model

To investigate the feasibility of the MHM model in multipath mitigation for a moving platform, we performed our shipborne experiments from two aspects. First, we checked the consistency of the derived MHM from static status and from moving status. Second, we examined the consistency of the derived MHM from different time intervals with different moving statuses. Inside the software all partial derivatives and estimated parameters are implemented and processed in a geocentric Cartesian coordinate system. Then, the estimated position and baseline parameters and tabled satellite positions were transformed to the topocentric coordinate system (TCS) based on the estimated positions of the master antenna. The nature of this transformation is rotational, so the baseline vector is not very sensitive to the accuracy of the estimated positions of the master antenna due to the ratio to the radius of the Earth. For example, one meter of error in the estimated position of the master antenna will only generate about 0.03″ rotational error. After this transformation the satellite positions are expressed as azimuth and elevation angles and the baseline vectors are expressed as yaw, pitch, and roll angles relative to the TCS. The next step is transformation from TCS to the carrier coordinate system (CCS) based on the baseline yaw, pitch, and roll angles relative to the TCS. After this transformation, all satellite positions are expressed as azimuth and elevation angles in CCS. Then, the residuals were allocated to grid cells according to the satellite position in CCS. To construct the MHM model, the abnormal residuals due to either poor observations or local effects should be identified and eliminated, and the grid cells containing a few residuals should be tested to avoid unreliable average values as being pointed out by [27]. Thus for the grid cells with more than 5 residuals, the 3-sigma rule was adopted to identify and exclude the outliers; for the grid cells with less than 5 residuals, we compare the root mean square (RMS) of these cells before and after multipath correction. If the RMS of a grid cell appears larger after multipath correction than uncorrected, the averaged value of this grid cell will not be used. After the outlier elimination procedure, for each grid cell we take the average of residuals to represent the multipath effects for this cell; for the cells without residuals, we leave them as zero. The derived values of all grid cells are listed as the MHM model.

The TCS system is defined as the x-axis towards local east, the y-axis towards local north, and the z-axis pointing upward. The origin of the coordinate system coincides with the location of the master antenna of the multiple antenna GNSS receiver system. In the TCS, the satellite azimuth angle is defined as the angle measured clockwise from the y-axis to the x-axis, and the elevation angle is defined as the angle measured upwards from the x-y plane. Thus, we obtain

(9) $$\theta_{azi}=\arctan\left(\frac{x_{tcs}}{y_{tcs}}\right),\;\theta_{ele}=\arctan\left(\frac{z_{tcs}}{\sqrt{x_{tcs}^{2}+y_{tcs}^{2}}}\right)$$

The suffix “*tcs*” means the satellite coordinate in the TCS, and the suffixes “*azi*” and “*ele*” represent the satellite azimuth and elevation angles in this coordinate system, respectively. The azimuth angle ranges from 0° to 360°, and the elevation angle ranges from −90° to 90°. In most ground moving platforms, we only see the satellite above the horizon, so the elevation angle ranges from 0° to 90°. Meanwhile, the baseline yaw angle is defined as the clockwise rotation angle from the topocentric north to east, ranging from 0° to 360°; the pitch angle is measured as the upwards angle from the topocentric horizontal plane, ranging from −90° to 90°; and the roll angle is measured from the topocentric vertical plane, inclining towards right as positive and ranging from −180° to 180°. Since a single baseline can only determine the yaw and pitch angles of platforms simultaneously, we can obtain the yaw and pitch angles in the TCS:

(10) $$\theta_{yaw}=\arctan\left(\frac{x_{1}}{y_{1}}\right),\;\theta_{pitch}=\arctan\left(\frac{z_{1}}{\sqrt{x_{1}^{2}+y_{1}^{2}}}\right)$$

where $\left(x_{1},y_{1},z_{1}\right)$ are the coordinates of the slave antenna (i.e., the baseline vector components) in the TCS, and the suffixes “*yaw*” and “*pitch*” represent the yaw and pitch angles relative to the TCS.

For a moving platform, we define the CCS system as the y-axis towards the front along the major symmetric axis of the platform, the z-axis pointing upward, and the x-axis towards the right of the platform. The origin coincides either with the cross between the major symmetric axis and the minor symmetric axis, or with the mass center of the platform. For convenience, we can let the origin coincide with the location of the master antenna of the multiple antenna GNSS receiver system, so that the transformation of the satellite position between TCS and CCS is purely rotation without translation. In this case, the transformation is realized by the formula

(11) $${\left[\begin{array}{c} \Delta x\\ \Delta y\\ \Delta z \end{array}\right]}_{ccs}=[\begin{array}{ccc} cos\gamma cos\psi +sin\xi sin\gamma sin\psi & -cos\gamma sin\psi +sin\xi sin\gamma cos\psi & -cos\xi sin\gamma \\ cos\xi sin\psi & cos\xi cos\psi & sin\xi \\ sin\gamma cos\psi -sin\xi cos\gamma sin\psi & -sin\gamma sin\psi -sin\xi cos\gamma cos\psi & cos\xi cos\gamma \end{array}]{\left[\begin{array}{c} \Delta x\\ \Delta y\\ \Delta z \end{array}\right]}_{tcs}$$

where the suffixes “*ccs*” and “*tcs*” represent the satellite coordinate in the CCS and TCS, respectively; and ψ,ξ,γ represent the yaw, pitch, and roll angles of the baseline in the TCS, respectively. We also define the satellite azimuth and elevation angles in the CCS similar to that in the TCS, the azimuth angle is measured clockwise from the major axis (y-axis) of the platform, and the elevation angle is measured upwards from platform plane (x-y plane), so that the formula is the same as Equation (9), except the “*tcs*” is replaced by the “*ccs*”.

## 3. Experimental Results 

Our shipborne tests consisted of three parts. The first part was carried out to verify the spatial repeatability of multipath effects in a quasi-static shipborne environment. In the second part, we performed a moving shipborne test to assess the feasibility of the MHM approach in a kinematic shipborne environment. Finally, we investigated the factors affecting the performance of MHM for implementation in real-time and kinematic shipborne multipath mitigation.

### 3.1. Static Shipborne Test

**Figure 1:**
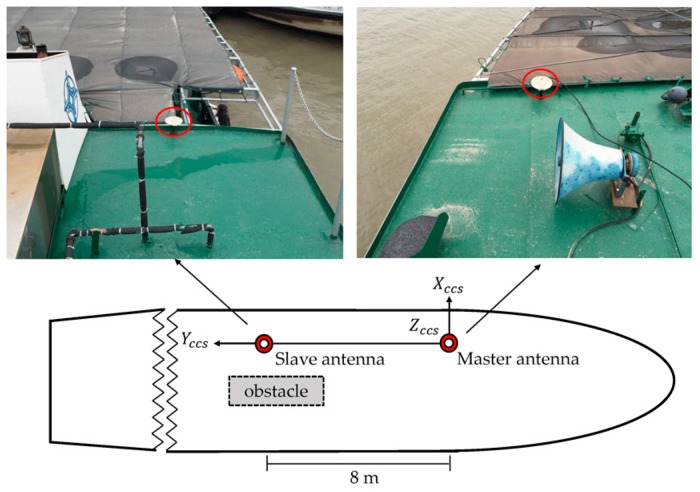
Antenna configuration and surrounding environment in static shipborne test. CCS: carrier coordinate system.

In the test, two antennas were mounted on the deck of the survey ship owned by Shanghai Dahua Surveying and Mapping Company. The distance between the two antennas was about 8 m (Figure 1). The baseline of the two antennas was parallel to the major symmetric axis of the ship. The antennas were mounted very low above the deck to ensure the primary multipath effects come from the ship itself. The nearby primary obstacle was close to the slave antenna and on one side of the baseline. Thus, significant baseline multipath effects were expected to occur at low elevation angles, and the azimuth angles were expected to be from about 60° to 160° in the CCS frame if there were satellite signals in these regions. These two antennas were connected to one Trimble BD982 GNSS receiver with a 1 Hz data sampling rate. The test started at 6 a.m. (Beijing time) on 28 January to 6 a.m. on 30 January 2015. The first 24 h and second 24 h are denoted by doy 28 and doy 29, respectively. During the two days, the ship docked at Dongjiadu wharf in Shanghai. However, the ship was not truly “static”, since the wind and waves always rocked the ship in small amplitudes. So, we still treated the baseline vector parameters as stochastic and allowed them to change with time. The results indicated that the maximum variation amplitude of yaw and pitch angles in the test reached 8° and 2°, respectively.

**Figure 2:**
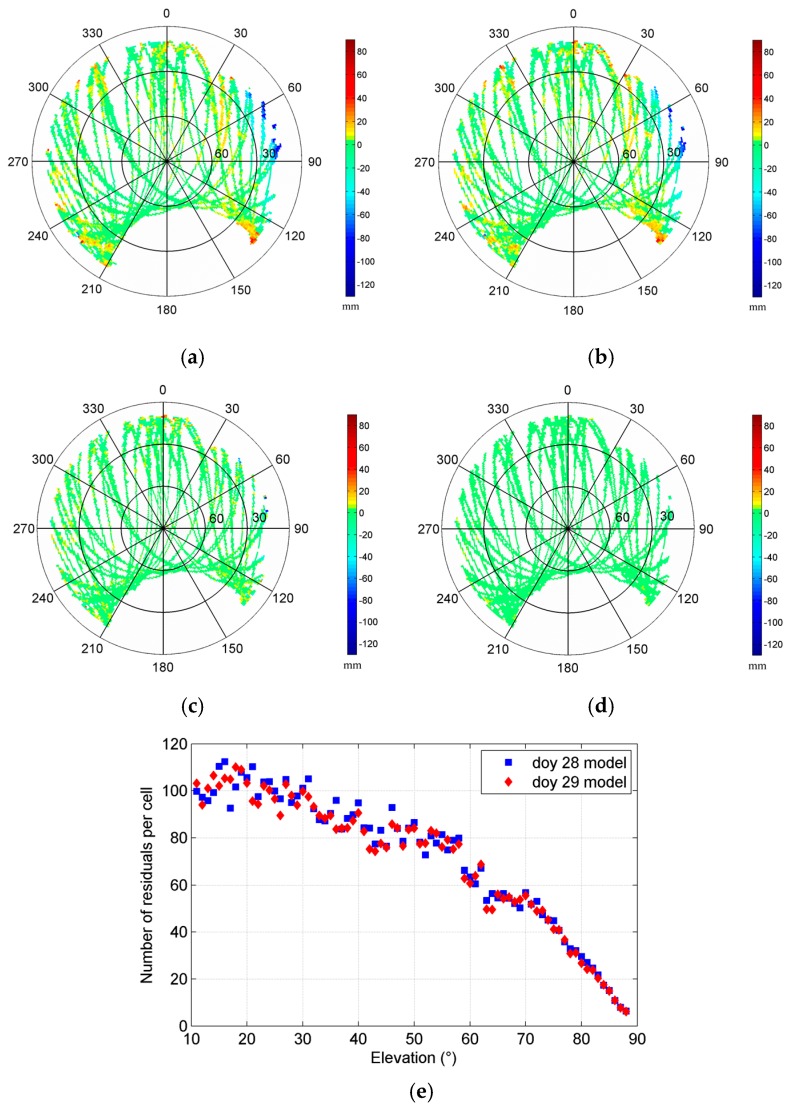
Sky trajectories of the multipath hemispherical map (MHM) grids from doy 28 (**a**) and doy 29 (**b**) (the strongest multipath effects caused by the nearby obstacle are distributed at the region where azimuth angles range from 60° to 120° and display in dark blue color). Sky trajectories of the doy 29 residual grids after multipath correction using the MHM model from doy 28 (**c**) and doy 29 (**d**). Number of residuals per cell for MHM model from doy 28 and doy 29 (**e**).

In this test, we constructed two 1° by 1° MHM models using the single-difference residuals from doy 28 and doy 29, respectively. Since two antennas can only determine yaw and pitch angles simultaneously, we set the roll angle to zero in Equation (11), assuming the variation of roll angle is small in a shipborne environment under mild waves. The sky trajectories of resulting MHM grids from doy 28 and 29 show very similar spatial pattern of multipath effects (Figure 2a,b). The multipath effects reached the 120 mm level in this test, and stronger multipath effects occurred at low elevation angles (15° < Ele < 30°), which agrees well with the environment surrounding the antennas. The statistics indicate that the doy 28 model covers about 83.29% of the grid cells of the doy 29 model. Figure 2e shows that the numbers of residuals per cell of the models from doy 28 and doy 29 are close and mostly more than 10. The sky trajectories of the doy 29 residuals’ grids, after using the MHM models from doy 28 and doy 29 (Figure 2c,d), show that the multipath effects are significantly mitigated after the multipath correction, and more than 90% of residual multipath effects are smaller than 6 mm. It also demonstrates that the assumption (the impact of roll angle variation under mild waves on MHM is minor) seems acceptable.

To quantitatively assess the performance of MHM for multipath mitigation in a static shipborne environment, we calculate the residual reduction percentage according to the formula $\left(1.0-r_{2}/r_{1}\right)\times 100\%$, where $r_{1}$ and $r_{2}$ are the RMS of residuals before and after multipath correction. First, we corrected the multipath effects in doy 29 using the MHM model generated from the same day. The results show that the residual reduction reaches 55.77%, indicating that the major portion of multipath effects stem from medium- and low-frequency variations in the quasi-static environment. Second, we remove the multipath effects in doy 29 using the MHM model generated from doy 28. The residual reduction drops to 39.89%, indicating the difference between the two MHM models. We will discuss the factors affecting the performance of MHM in detail in Section 3.3.

**Figure 3:**
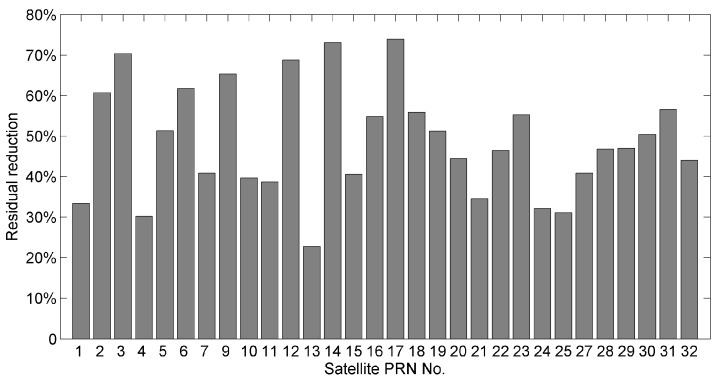
Residual reductions of doy 29 for different satellites after multipath mitigation using the model generated from the same day.

**Figure 4:**
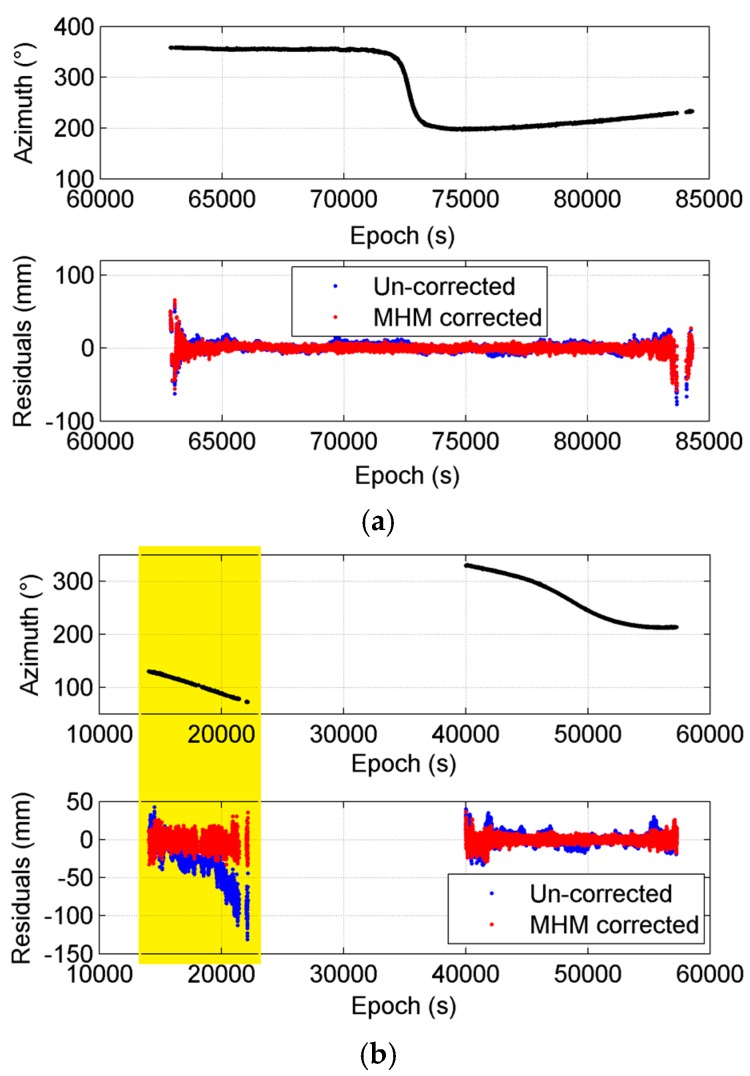
Comparison of residual series together with azimuth angles between satellite G13 (**a**) and G03 (**b**). The region where azimuth angles range from 60° to 160° is highlighted in yellow.

Then, we computed the residual reduction per satellite. Figure 3 displays the doy 29 residual reductions for different satellites using the MHM model generated from the same day. The results show that the residual decreases significantly after multipath mitigation (mostly > 30%). Furthermore, it can be seen that residual reductions vary by satellites. The largest residual reduction reaches 73.92%, and the smallest is 22.77%. In order to explain the reason for different performances, we compared the residual time series together with the azimuth angles between satellites G13 (22.77% reduction) and G03 (70.38% reduction) (Figure 4). The comparisons demonstrate that the amplitude of the original residual series (blue curves) of G13 (top panel) is much smaller than that of G03 (bottom panel). In addition, the residual series of G03 after MHM multipath correction (red curves) are much flatter than that of G13. Two plausible causes are revealed by the comparisons. First, our reduction percentage formula does not exclude observation noise, which cannot be corrected by the multipath model. If the observation noise levels are similar for each satellite, the residual reduction percentages are expected to be lower for the satellites with smaller residual amplitudes. Second, our current MHM model is constructed by simply taking the average for each grid cell, and such a constructed MHM model is inclined to the satellites with large multipath effects. In particular, the G03 residual series have a segment with azimuth angles ranging from 60° to 120° (Figure 4b), which is linked to the obstacle near the slave antenna. We specifically calculated the residual reduction for this region (60° < Azi < 120°); it increased to 76.38%.

**Figure 5:**
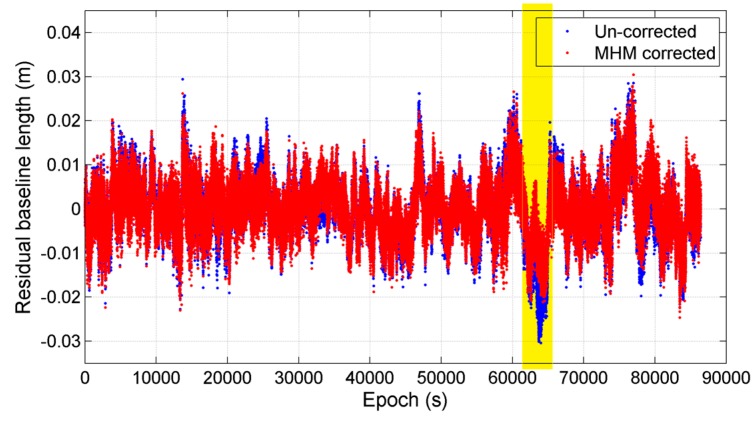
Comparison of residual baseline length series before and after MHM multipath correction in static test, where the “residual” represents that the average baseline length is subtracted from both baseline length series. The time period (epoch 63,000–65,000 s) when significant improvement occurred is highlighted with yellow.

Finally, we compared the baseline length solutions with and without applying MHM multipath correction. Since the baseline length is constant during the test, the average of baseline length solutions is taken as “ground truth” (about 8.1024 m). Figure 5 shows the doy 29 residual baseline length series before and after multipath correction using the model from doy 28, where the nominal value is subtracted from the original baseline length series. The statistics shows that the mean deviations of baseline length solutions before and after multipath correction agree to within 0.1 mm, and the RMS of baseline length deviations is reduced by 10.47% after multipath mitigation. 

**Figure 6:**
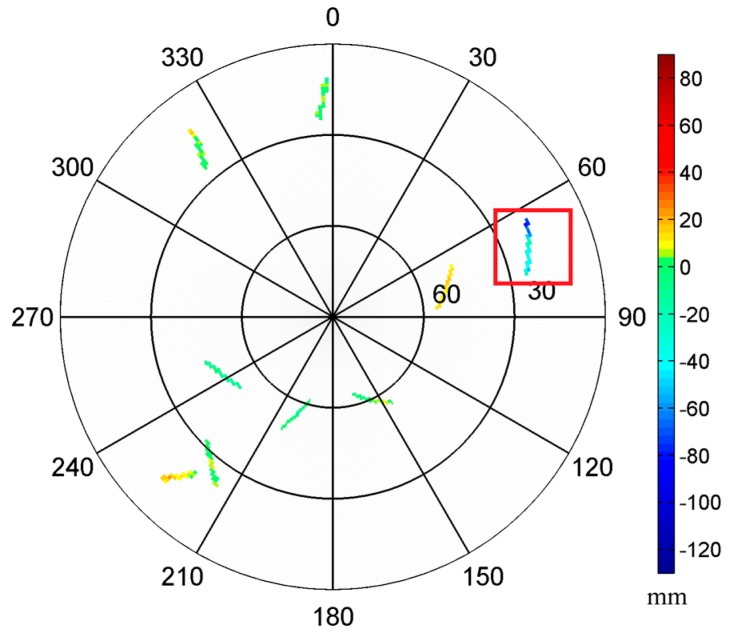
Sky trajectories of the multipath hemispherical map (MHM) grids using the residuals at time period (epoch 63,000–65,000 s). The strong multipath effect caused by the obstacles nearby are marked with red box.

**Figure 7:**
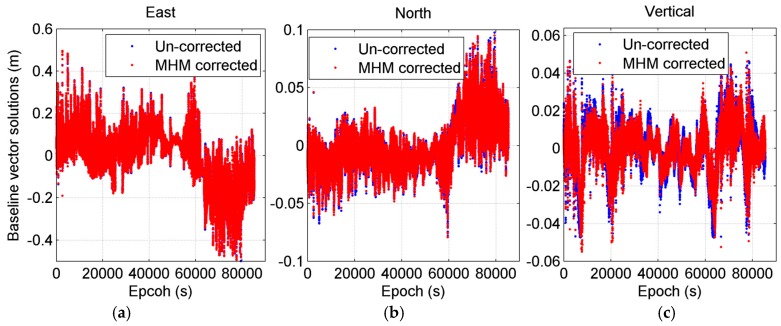
Baseline vector solutions series for the east (**a**); north (**b**); and vertical (**c**) components before and after MHM multipath correction. The average baseline solutions are subtracted from all original baseline solutions series.

The results also show that the significant improvement in the baseline solutions occurred during the period from 63,000 to 65,000 s (as marked in Figure 5). The sky trajectories of the residuals within this period (Figure 6) show that both the deviation and improvement of baseline length solutions are primarily caused by the strong multipath effect at the region (60° < Azi < 90°, as marked with red box), which correspond to the nearby obstacles in the shipborne environment (Figure 1). We also compared the baseline vector solutions before and after MHM multipath correction (Figure 7), and the results indicate that the MHM multipath corrections mostly affect the vertical component of baseline vector solutions.

### 3.2. Kinematic Shipborne Test

**Figure 8:**
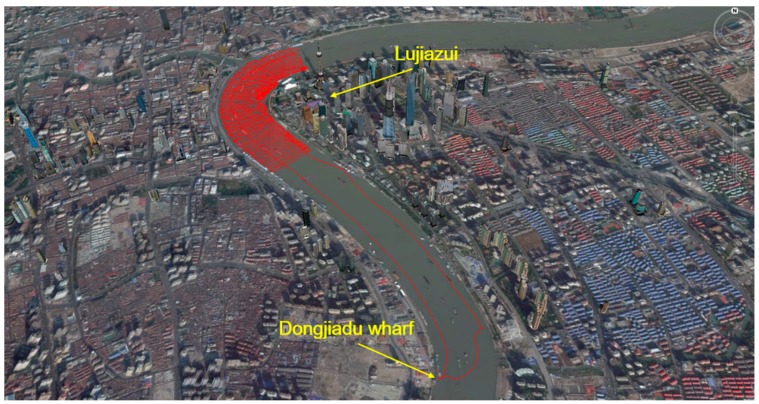
The trajectory of the kinematic shipborne test.

In this test, we adopted the same GNSS receiver, and kept the locations of two antennas in the ship unchanged. This test was carried out on doy 32, 2015, and the trajectory is shown in Figure 8. At first, the ship docked in the Dongjiadu wharf for about one hour, then left to Lujiazui along the east side of the Huangpu River. When sailing near to Lujiazui, it began to repeatedly sail perpendicular to the flow direction in order to carry out underwater surveys until it reached Lujiazui. Finally, it returned to Dongjiadu wharf along the west side of river. The whole voyage lasted about 8 h. The data sampling rate was 1 Hz.

In order to evaluate the performance of the MHM in the kinematic shipborne environment, we applied the MHM multipath correction in two cases: first, we corrected the multipath effects using the 8 h MHM model, which represented the ideal case (all grid cells are fully covered) and provided a reference for comparison. Second, we divided the 8 h into two sections: the first five hours were used to construct the multipath correction map, which was applied in last three hours for multipath mitigation.

#### 3.2.1. Performance Evaluation of the 8 h Model

**Figure 9:**
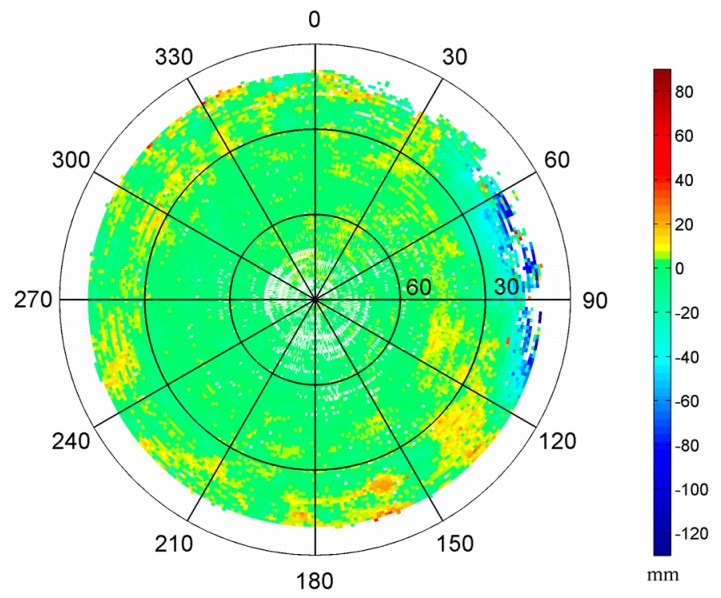
Sky map of the MHM grids of kinematic shipborne test.

At first, we constructed 1° by 1° grid MHM model using the residuals from the kinematic test. As shown in Figure 9, in the CCS frame, the rotation of the ship smears the trajectories of satellites in the sky; nearly all grid cells above 10° elevation angle are covered. The multipath effects display circular-shaped belts, which are generated by the combination of the movements of satellites and ship. Meanwhile, they show similar spatial distribution as the sky maps of the static test. The consistency of derived MHM models from static and kinematic status indicates that the predominant multipath effects in the shipborne environment arise from the structure of the ship, which exhibits the feasibility for the MHM approach in the kinematic shipborne environment.

**Figure 10:**
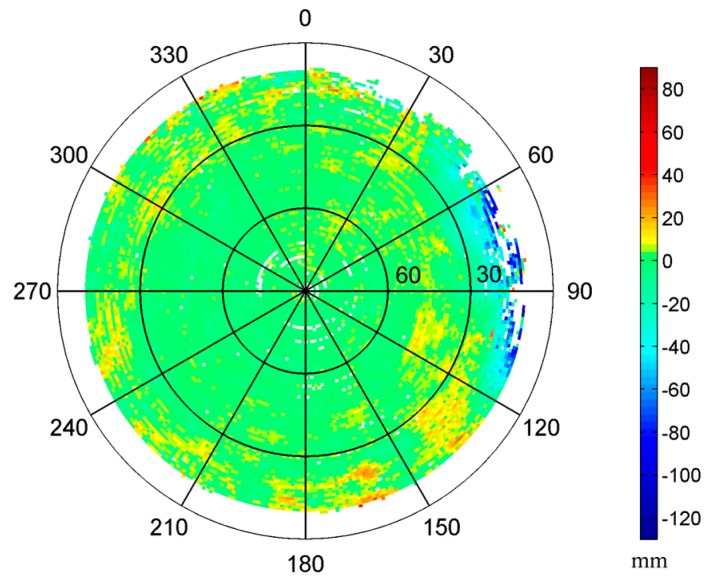
Sky map of the MHM grids with congruent cells in kinematic shipborne test.

After correcting the multipath effects using the 8 h MHM model, the residual reduction is reduced to 48.74%. There are some empty grid cells at high elevation angles due to missing orbital coverage. We tried to use the congruent cells [27] to construct the MHM model, where the shape and size of each grid cell are similar. We set the cell size of elevation angle to 1°, while the size of azimuth angle is 1° at Ele = 0° and then increases with the higher elevation angles based on the equal grid cell area rule. Figure 10 shows the sky map of MHM grids with congruent cells. Compared with the map with fixed azimuth resolution (Figure 9), the spatial distribution of multipath effects is similar, while the model with congruent cells is nearly fully covered at high elevation angles.

**Figure 11:**
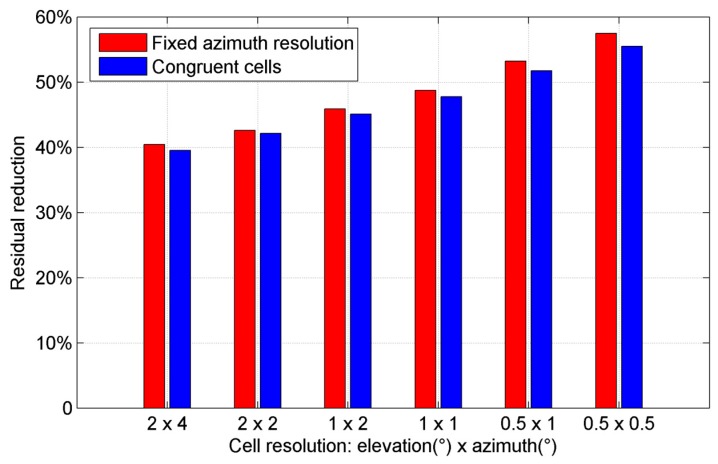
Comparison of residual reductions after using the 8 h MHM model with fixed azimuth resolution and congruent cells in kinematic shipborne test, where the resolution of azimuth angle means the smallest resolution occurring at Ele = 0°.

Then, we compared the residual reductions after using the 8 h MHM models with fixed azimuth resolution and congruent cells (Figure 11). The results show that the residual reductions from the two models are very close, and the best results are achieved by using the resolution of 0.5° by 0.5° for both models; this agrees with the conclusion in [27].

**Figure 12:**
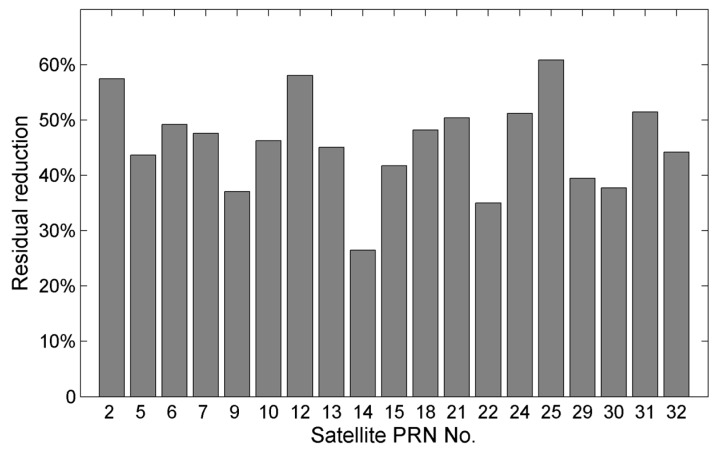
Residual reductions for different satellites after multipath mitigation using the 8 h MHM model.

Given the close performances of the two models, the MHM model with fixed azimuth resolution is adopted in the following sections. Figure 12 shows the residual reductions for different satellites using the 8 h MHM model. After the MHM multipath mitigation, most residual reductions exceed 30%; the largest residual reduction reaches 60.82% and the smallest is 26.49%.

**Figure 13:**
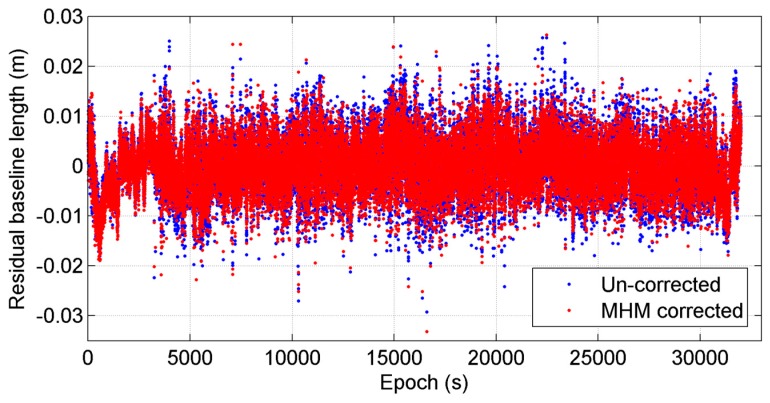
Comparison of residual baseline length series before and after MHM multipath mitigation, where the “residual” represents that the average baseline length is subtracted from original baseline length series.

Finally, we compared the baseline length solutions before and after MHM multipath correction. Figure 13 shows the residual baseline length series before and after multipath correction using the 8 h model, the average of baseline length series (about 8.1024 m) is subtracted from both baseline length series. The results indicate that the mean deviations of baseline length solutions before and after multipath mitigation are both close to zero, and the RMS of baseline length deviations is reduced by 9.20% after multipath correction.

#### 3.2.2. Performance Evaluation of the 5 h Model

**Figure 14:**
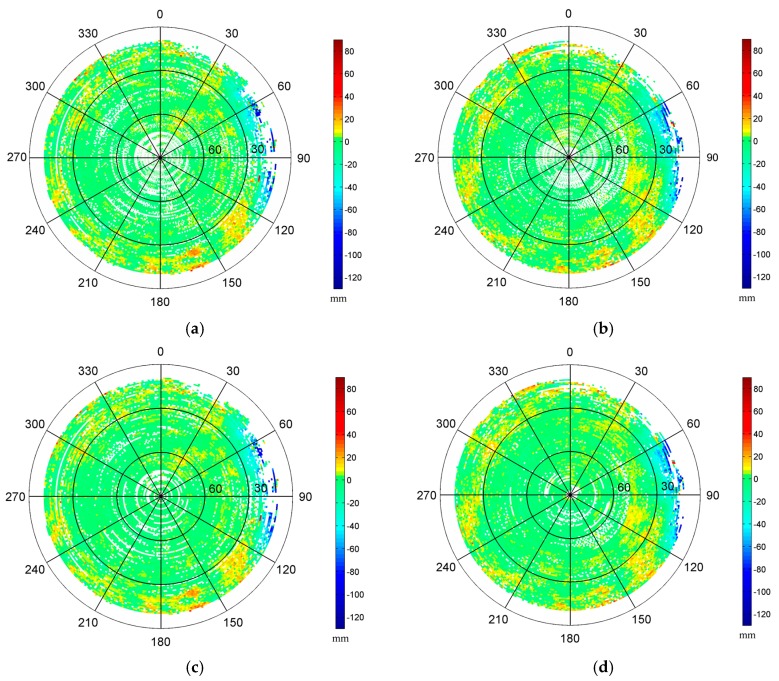
Sky map of the MHM grids from first five hours (**a**) and last three hours (**b**) in kinematic shipborne test. Sky map of the MHM grids with congruent cells from the first five hours (**c**) and last three hours (**d**) in kinematic shipborne test.

In this case, the first five hours were used to establish the grid MHM model, and the last three hours were applied with multipath correction using the first 5 h MHM model. The unequal division was designed to ensure enough spatial coverage of the multipath correction model. At first, we constructed two 1° by 1° grid MHM models using the residuals from the first five hours and last three hours (Figure 14a,b). The first 5 h model can cover about 79.32% of the grid cells of the last 3 h model. With the congruent cells model, the coverage increases to 88.37% (Figure 14c,d), and the residual reduction percentage is similar to the model with fixed azimuth resolution. The sky maps of two sections clearly show similar spatial distribution, and the maximum multipath effects are approximately 120 mm. The relatively larger multipath error occurs at the same region in two sky maps (15° < Ele < 30°, 60° < Azi < 160°). The consistency of the resulting MHM models from different intervals with different moving statuses indicates that the dominant multipath effects in this case stem from the ship itself, and that the influence from the surrounding environment (such as the distant structures) is minor.

**Figure 15:**
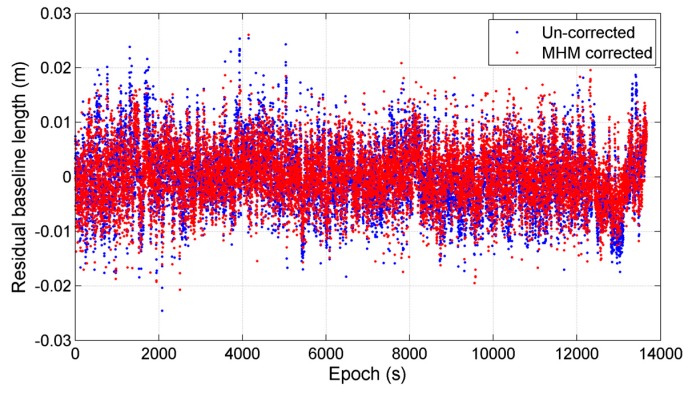
Comparison of residual baseline length series before and after MHM multipath mitigation in kinematic test, where the “residual” represents that the average baseline length is subtracted from original baseline length series.

Then, we mitigated the multipath effects in the last three hours using the first 5 h model. The results show that the residual reduction drops to 21.91%. We will discuss the causes of the drop of the residual reduction in the next section. Finally, we compared the baseline length solutions before and after MHM multipath correction to evaluate the performance of the MHM approach. Figure 15 compares the residual baseline length series of last three hours before and after multipath correction using the first 5 h model, where the average of baseline length series (about 8.1027 m) is subtracted from the baseline length series. Statistics indicates that the mean deviations of baseline length solutions before and after multipath mitigation are also close to zero, and the RMS of baseline length deviations is reduced by 10.57% after multipath correction. Moreover, we also corrected the multipath effects in the last three hours using the 8 h model, and the RMS of baseline length deviations is lowered by 12.70%. 

### 3.3. Influence Factors Analysis 

Although the feasibility of the MHM approach in real-time and kinematic shipborne environments has been demonstrated, from the perspective of practical application, the factors affecting the performance of MHM still deserve in-depth investigations.

#### 3.3.1. Observation Noise

**Figure 16:**
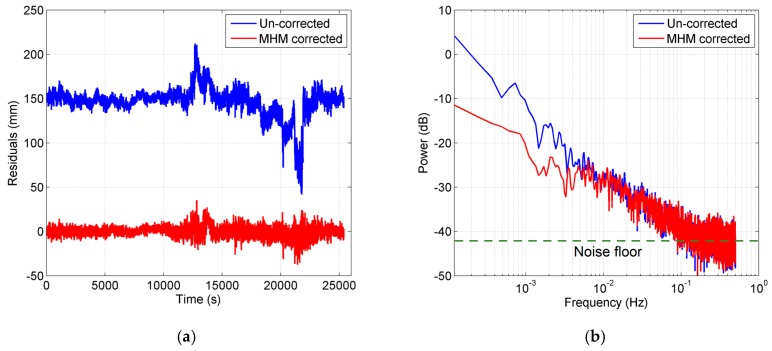
(**a**) Residual series of satellite G12 before and after multipath mitigation in static test. Offsets (150 mm) are assigned to the uncorrected residuals; (**b**) power spectrum density of residual series. Blue curve: before multipath mitigation. Red curve: after MHM model mitigation. The noise floor is marked with the dotted green line.

**Figure 17:**
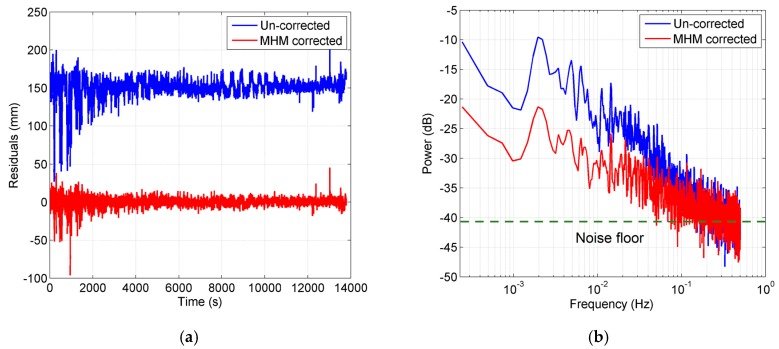
(**a**) Residual series of satellite G12 before and after multipath mitigation in kinematic test. Offsets (150 mm) are assigned to the uncorrected residuals; (**b**) power spectrum density of residual series. Blue curve: before multipath mitigation. Red curve: after MHM model mitigation. The noise floor is marked with the dotted green line.

Figure 16a shows the residual series of satellite G12 before and after the multipath correction in the static test. The uncorrected residual series shows low-frequency patterns and high-frequency variations. After the multipath mitigation, the low-frequency patterns are obviously eliminated and the series becomes flat, while the high-frequency variations still exist. For the kinematic test, the residual series before and after multipath mitigation has a similar performance, as shown in Figure 17a. We also performed power spectrum analysis for these four residual series (Figure 16b and Figure 17b). The results illustrate that the low-frequency multipath effects are removed significantly after MHM is applied, while the spectral power in the high-frequency band remains almost unchanged after multipath mitigation. These results agree well with the conclusions in [28]. 

The residuals after multipath mitigation consist of two parts: the uneliminated multipath effects and the observation noise. The part with slope in power spectra plot (lower than 0.1 Hz) belongs to the uneliminated multipath effects plus noise floor from the observation noise, while the flat part in the power spectra plot (higher than 0.1 Hz) is regarded as primarily from the observation noise, which cannot be eliminated by the MHM model. Since we assume that the observation noise is white noise, the power spectrum of white noise is statistically flat, which matches the power spectra higher than 0.1 Hz. Meanwhile, the comparison between Figure 16b and Figure 17b also demonstrates that the observation noise in the kinematic case is significantly higher than that in static case.

According to our assumption, the signal and observation noise is uncorrelated. The observation noise can be easily isolated in the frequency domain, thus we attempted to quantitatively assess the influence from observation noise on residual reduction. Based on Parseval’s theorem, the total power of a discrete time series calculated in the time domain equals the total power calculated in the frequency domain. For a discrete time series, the Parseval’s relationship is expressed as:

(12) $$\overset{N-1}{\underset{n=0}{\sum }}{\left|x\left[n\right]\right|}^{2}=\frac{1}{N}\overset{N-1}{\underset{k=0}{\sum }}{\left|X\left[k\right]\right|}^{2}$$

where the x on left-hand side represents the discrete time series; X on right-hand side represents the discrete Fourier transform (DFT) of x, both of length N. Thus the mean power of the observation noise can be calculated in the frequency domain. Since the power spectrum of white noise should be a constant for the whole frequency band, the flat part in the power spectrum (higher than 0.1 Hz) is regarded as being from the observation noise, thus the noise floor of the power spectrum represents the contribution of the observation noise (as marked in Figure 16b and Figure 17b). As for the slope part, the observation noise still exists, but is mixed with the residual multipath effect. Thus in this paper, we calculated the mean power of the noise floor (spectrum higher than 0.1 Hz) as the mean power spectrum of the observation noise, which is equivalent to the mean power of the observation noise in time domain according to the Parseval’s theorem. Then we subtracted the mean power of the observation noise to obtain the power of residual multipath for both residual series before and after multipath correction. The residual reduction before and after the observation noise correction can be expressed as:

(13) $$R=1-\frac{r_{2}}{r_{1}}=\frac{\sqrt{X_{s1}^{2}+X_{n}^{2}}-\sqrt{X_{s2}^{2}+X_{n}^{2}}}{\sqrt{X_{s1}^{2}+X_{n}^{2}}}$$

(14) $$R_{c}=1-\frac{r_{2}}{r_{1}}=\frac{\sqrt{X_{s1}^{2}}-\sqrt{X_{s2}^{2}}}{\sqrt{X_{s1}^{2}}}=\frac{X_{s1}-X_{s2}}{X_{s1}}$$

where $X_{s1}^{2},X_{s2}^{2}$ represent the residual multipath variance before and after multipath correction, respectively, and $X_{n}^{2}$ represents the noise variance. It can be easily proven that the residual reduction after observation noise correction is higher than before the correction. In our case, the residual reductions for the static test are enhanced from 55.77% and 39.89% to 58.46% and 41.68%, respectively, and for the kinematic test it is raised from 48.74% and 21.91% to 52.77% and 24.51%, respectively. Additionally, the observation noise in the kinematic test was higher than in the static test with a maximum of $2.21\times 10^{-2}$ cycles and $1.49\times 10^{-2}$ cycles at L1 frequency, respectively. Note that the assumption (observation noise is regarded as white noise) provides a lower bound of the power of the observation noise since the real observation noise likely also contains a flicker noise component [31].

#### 3.3.2. Coverage

A good coverage of the grid MHM models is the key to success in multipath mitigation. However, the full coverage of MHM models is not always available, especially for kinematic applications. We explored the influence of model coverage on the performance of the MHM approach.

Table 1 compares the residual reductions after using the models with different coverage in the static test (the doy 29 MHM model) and kinematic test (the 8 h MHM model), respectively. Those MHM models with different coverage are established by randomly selecting the grid cells in a certain ratio from the model with full coverage. The results indicate that the full coverage of the grid MHM model guarantees the best performance of multipath mitigation. With the decrease in model coverage, its performance is worsened. Especially for the moving platforms, the incomplete coverage of the grid MHM model will obviously undermine the effectiveness of the MHM approach to multipath correction. Additionally, the time length of observation for MHM modeling is related to grid coverage and sample redundancy, hence longer time length is recommended. According to the sky maps of the kinematic test, the variation of the multipath effects is smooth in adjacent grid cells. Thus, for the grid cells without any residuals, two spatial interpolation approaches—including inverse distance weighting [32] and Kriging interpolation [33]—were adopted to obtain the valid interpolation results to replace zero. The results show that the residual reductions after using these two approaches are very close to 24.94% and 25.12%, respectively (within 1.5° search radius), which is an increase by approximately 14% compared with the uninterpolated model. Thus, compared to the uninterpolated model, the spatial interpolation approaches are necessary to remedy the performance degradation due to the sharp movement of the ship and limited spatial resolution.

#### 3.3.3. Sparse Resolution

According to the statistical results, there are usually hundreds, even thousands, of residuals falling inside one grid cell, and the average of all the residuals in same grid cell is used as the multipath error in this grid cell. Thus, 1° by 1° grid cell resolution is still too sparse and misses the high-frequency multipath effects. The far-field multipath effects generate more high-frequency variations, and near-field effects show more low-frequency variations, hence the far-field multipath effects caused by distant structures cannot be effectively eliminated. This problem has been demonstrated [28] and requires deep investigation to improve the MHM model for high-frequency multipath mitigation. We will further expand our study on that in future work.

#### 3.3.4. Roll Angle

Due to the limitation of our receiver and shipborne availability, we were only able to carry out the experiments using a dual antenna GNSS receiver with a common clock. Thus, only the yaw and pitch angles could be determined, and we had to set the roll angle to zero in Equation (11) for the TCS-to-CCS transformation. The errors from such an omission are two-fold. First, such an omission generates inaccurate satellite elevation and azimuth angles in CCS, and therefore builds an inaccurate MHM model. Second, for the same reason, the residuals could be related to incorrect grid cells during the multipath correction, and could therefore cause a correction error. The level of grid misalignment is proportional to the degree of the roll angle. In our experiments, the impact of a missing roll angle on the multipath mitigation was much more serious in the kinematic environment than in the static environment, because during the voyage, the interaction between the moving ship and the waves produced much larger roll angles. This is one explanation for the large drop of residual reduction in the kinematic experiment.

## 4. Conclusions

Multipath effects are omnipresent in GNSS positioning and attitude determination. In GNSS kinematic positioning the multipath emerges as one of the primary error sources. While the RTK technique successfully eliminates most systematic errors, multipath error still remains. Theoretical investigation indicates that the spatial-repeatability-based multipath mitigation approaches are not only suitable for static positioning, but also for kinematic positioning when the dominant multipath effects arise from the moving platform itself. Previous studies have demonstrated the feasibility of the MHM approach in static environments. This paper extends this method to kinematic platforms, where the dominant multipath effects come from the carrier body itself. The feasibility of the MHM approach in real-time and in a kinematic shipborne environment was validated through static and kinematic shipborne tests. Experimental results indicate that after MHM multipath correction the RMS of baseline length deviations are reduced by 10.47% and 10.57%, and the RMS of residual values are reduced by 39.89% and 21.91% for the static and kinematic test, respectively. Additionally, the residual reductions for different satellite were calculated, showing that the residual reductions vary by satellites: the largest residual reduction reaches 73.92% and the smallest reduction is 22.77% in the static test, with similar results for the kinematic test. These results verify the feasibility of MHM for multipath mitigation in kinematic shipborne environments, but also indicates that the factors affecting the performance of MHM still require further analysis. Power spectrum analysis was used to analyze the residual series from time and frequency domains. The results show that MHM is more effective in mitigating low-frequency multipath effects, while the high-frequency multipath effects (indistinguishable from observation noise) still exist. After observation noise correction, the residual reductions grow to 41.68% and 24.51% in static and kinematic tests, respectively, which illustrates that the influence on the performance of MHM in kinematic shipborne environments is not ignorable. Besides, the effect from spatial coverage and resolution on the performance of MHM were also investigated to provide a guideline for future work. 

The MHM approach is an effective tool for multipath mitigation in kinematic shipborne environments. First, the number of grids is much smaller than the coefficients of the spherical harmonic model [24] under the same spatial resolution. Second, the MHM model appears to be much easier to implement in real-time multipath mitigation for moving platforms, because during the implementation the MHM approach simply inquires the value of the grid cell corresponding to the elevation and azimuth angles of the satellite. The harmonic approach, however, must calculate spherical harmonics of all degrees and orders, and then performs summation at every epoch for each satellite. Third, it provides an effective way to construct the MHM model covering all grid cells. Based on our experiments such an MHM model can be constructed within one day by rotating the ship. Additionally, the MHM approach can be applied to other GNSS observations in principle, however, different models are required for the observations at different frequencies. However, current performance of MHM in a kinematic shipborne environment is still unsatisfactory and requires further improvement. First, full attitude angles (yaw, pitch, and roll angles) should be used to construct the MHM model and to perform multipath correction. This means at least three GNSS antennas are required for full attitude determination. Second, the constructed MHM model must cover all grid cells. Based on our experiments, missing grid cells will downgrade the effectiveness of the MHM approach significantly. Additionally, performing spatial interpolation from surrounding grid cells is helpful to remedy the performance degradation due to the incomplete coverage. Third, simply taking a mean value for each grid cell seems to be inadequate. Extensive investigations are required to enhance the capability of the MHM model to mitigate the high-frequency multipath effect.

## Figures and Tables

**Table 1:** Residual reduction after multipath mitigation using MHM models with different coverage for static and kinematic tests.

	Coverage	100%	80%	50%	30%
Residual Reduction					
**Static Shipborne Test**		55.77%	51.38%	40.90%	27.10%
**Kinematic Shipborne Test**		48.74%	44.76%	35.00%	24.96%
